# Persistent Focal Myocardial ^18^F-FDG Uptake Discordant with CMR Oedema on Hybrid PET/MRI Six Months After Acute Myocarditis

**DOI:** 10.3390/diagnostics16182941

**Published:** 2026-09-11

**Authors:** Guillaume Reverdito, Hichem Sakhi

**Affiliations:** 1 Department of Radiology, Hôpital Henri Mondor, 94000 Créteil, France; 2 Department of Radiology, Hôpital Marie Lannelongue, Fondation Paris-Saint Joseph, 92360 Le Plessis-Robinson, France

**Keywords:** myocarditis, hybrid PET/MRI, ^18^F-FDG, cardiac magnetic resonance, myocardial inflammation

## Abstract

**Background**: Hybrid positron emission tomography/magnetic resonance imaging (PET/MRI) enables simultaneous assessment of myocardial metabolism and CMR tissue characteristics. We investigated whether integrated ^18^F-FDG PET/MRI could identify persistent metabolic abnormalities approximately 6 months after acute myocarditis, including abnormalities discordant with concomitant CMR oedema markers. **Methods**: In this retrospective single-centre study, 20 consecutive patients with a previous episode of acute myocarditis underwent integrated cardiac ^18^F-FDG PET/MRI approximately 6 months after the acute event. Focal myocardial FDG uptake, myocardial SUVmax and SUVmean, target-to-background ratios, T2 mapping, late gadolinium enhancement (LGE), and ventricular function were assessed within the hybrid examination. **Results**: Hybrid PET/MRI was performed at a median of 202 days (interquartile range [IQR], 173–221) after the acute episode. Four patients (20%) had non-diagnostic PET because of inadequate myocardial glucose suppression. Among 16 evaluable patients, four (25%) remained PET-positive with focal myocardial FDG uptake. Their myocardial SUVmax was 2.85 (IQR, 2.58–3.25) versus 1.65 (IQR, 1.38–1.80) in PET-negative patients (*p* = 0.001), and the myocardium-to-blood-pool ratio was 1.03 (IQR, 1.00–1.19) versus 0.82 (IQR, 0.75–0.86), respectively (*p* = 0.003). None of the four PET-positive patients had myocardial oedema on concomitant T2 mapping, whereas all had persistent LGE. One PET-negative patient showed persistent CMR oedema. **Conclusions**: Integrated PET/MRI identified persistent focal metabolic abnormalities in 25% of evaluable patients approximately 6 months after acute myocarditis, despite absent concomitant CMR oedema. Although the cohort is small and contemporary longitudinal clinical characterisation is limited, these hybrid-imaging findings support further investigation of PET/MRI as a tool for phenotyping the post-acute phase of myocarditis.

## 1. Introduction

Myocarditis is an inflammatory disease of the myocardium with heterogeneous etiologies, presentations, and outcomes [1,2,3,4,5,6]. Although the clinical course is frequently favourable, persistent myocardial inflammation may contribute to adverse remodelling, ventricular dysfunction, and progression toward inflammatory dilated cardiomyopathy [3,4,5].

Cardiac magnetic resonance (CMR) has a central role in the non-invasive diagnosis of myocarditis. The updated Lake Louise approach combines markers of myocardial oedema and non-ischemic myocardial injury [7]. ^18^F-FDG PET provides complementary metabolic information and is established in other inflammatory cardiac conditions, particularly cardiac sarcoidosis [8]. Hybrid PET/MRI is especially attractive because metabolic activity, ventricular function, myocardial oedema, and tissue injury can be assessed within a single integrated examination [9]. Previous reports have demonstrated the feasibility of integrated PET/MRI in acute myocarditis [10,11]. In patients presenting with myocardial injury and non-obstructive coronary arteries, myocarditis also represents an important differential diagnosis within the broader MINOCA spectrum [12,13].

Quantitative CMR mapping has improved characterisation of myocardial inflammation. Reference values for myocardial T1 and T2 at 1.5 T and 3 T and standardised recommendations for mapping techniques have been reported [14,15], while T2 mapping has been specifically investigated for myocardial oedema assessment [16]. Abnormal T2 mapping has also been associated with clinical outcome in suspected acute myocarditis [17], and T1/T2 mapping has shown value across inflammatory myocardial diseases and in distinguishing acute from more chronic phenotypes [18,19]. More recently, practical recommendations from the European Society of Cardiovascular Radiology have emphasised the complementary roles of CMR and PET in acute myocarditis and chronic inflammatory cardiomyopathy [20]. A 2025 systematic review of hybrid PET/MRI in inflammatory cardiac diseases further highlighted the incremental diagnostic contribution of hybrid imaging, particularly when standalone imaging findings were equivocal [21]. Nevertheless, evidence regarding simultaneous PET/MRI during longitudinal follow-up after a documented episode of acute myocarditis remains limited.

The aim of this study was therefore to use integrated ^18^F-FDG PET/MRI approximately 6 months after acute myocarditis to characterise persistent metabolic activity and its relationship with simultaneously acquired CMR markers, with particular attention to PET/CMR discordance during the post-acute phase.

## 2. Materials and Methods

### 2.1. Study Population

This retrospective, observational, single-centre study was conducted at our centre. The study was conducted in accordance with the Declaration of Helsinki, and approved by the CERIM Ethics Committee for Medical Imaging Research (IRB CRM-1907-026; 7 July 2019). Between March 2018 and November 2019, 20 consecutive patients with a previous episode of acute myocarditis underwent cardiac PET/MRI during follow-up.

At the acute stage, patients underwent clinical assessment, laboratory testing including high-sensitivity troponin, C-reactive protein and NT-proBNP, transthoracic echocardiography, and CMR. Significant coronary artery disease was systematically excluded by coronary computed tomography angiography or invasive coronary angiography. The diagnosis of acute myocarditis at the index presentation was based on a compatible clinical presentation, evidence of myocardial injury, and CMR findings fulfilling the Lake Louise Criteria. At baseline, myocardial oedema was present in all patients (100%). During follow-up, five patients (25%) received heart-failure therapy and three received NSAIDs/colchicine (15%) during 3 months. Furthermore, 17 patients (85%) received no specific anti-inflammatory or immunosuppressive treatment.

### 2.2. PET/MRI Acquisition

Examinations were performed on an integrated 3-T PET/MRI system (Biograph mMR, Siemens Healthineers, Erlangen, Germany). PET images were acquired 60 min after intravenous administration of ^18^F-FDG (mean injected activity, 340.5 MBq [297–384 MBq]) using a 344 × 344 matrix and reconstructed in 3D with a 2 × 2 × 2 mm^3^ voxel size. MR-based attenuation correction was performed using a four-compartment attenuation map derived from Dixon water- and fat-only images, with segmentation into background, lung, fat, and soft-tissue compartments. Patients followed a low-carbohydrate, high-fat diet for 24 h before imaging to suppress physiological myocardial glucose uptake. This was followed by a fasting period of at least 12 h. Blood glucose was measured before tracer administration. No intravenous heparin was administered. Patients received approximately 3 MBq/kg of ^18^F-FDG intravenously. PET images were acquired approximately 60 min after intravenous ^18^F-FDG administration. Injected activity and body weight were recorded in the main study database. Quantitative myocardial and background PET measurements, including SUVmax, SUVmean, blood-pool activity, aortic activity, and target-to-background ratios, were retrieved from a separate dedicated PET quantification dataset generated for the study.

The CMR protocol included cine steady-state free-precession imaging for ventricular function, native T1 mapping, T2 mapping, post-contrast T1 mapping, and LGE imaging after gadolinium administration (Dotarem, Guerbet, Villepinte, France).

### 2.3. Image Analysis

Hybrid PET/MRI examinations were jointly interpreted by an experienced cardiovascular radiologist with more than 5 years of experience and a nuclear medicine specialist. PET was considered positive when focal pathological myocardial ^18^F-FDG uptake was present in at least one myocardial segment. PET positivity was determined visually based on focal myocardial uptake considered abnormal in distribution and intensity after adequate suppression of physiological myocardial glucose uptake. Quantitative SUV and target-to-background measurements were used to characterise FDG uptake but were not used to define PET positivity. Focal uptake was distinguished from incomplete physiological suppression based on its discrete regional distribution, whereas diffuse or heterogeneous physiological uptake patterns were considered indicative of inadequate suppression and classified as non-diagnostic. Quantitative PET analysis used the dedicated PET quantification dataset and included myocardial SUVmax and SUVmean, blood-pool and aortic background activity, and myocardium-to-blood-pool and myocardium-to-aorta ratios. PET and CMR data were acquired during the same integrated examination, allowing direct comparison of metabolic activity with simultaneously acquired CMR tissue-characterisation markers.

Persistent myocardial oedema was assessed using T2 mapping according to local 3-T reference values and established mapping methodology [14,15,16]. Persistent myocardial oedema was assessed using T2 mapping acquired at basal/mid/apical levels in short-axis view and the four-chamber view (four slices) and analysed according to the 17-segment model. Myocardial oedema was defined using the local 3-T reference threshold of >55 ms, according to local 3-T reference values and established mapping methodology. The spatial coverage of T2 mapping was reviewed against the distribution of focal FDG uptake to ensure direct assessment of PET-positive myocardial regions. Persistent LGE in the absence of oedema was not considered evidence of active myocarditis. LVEF, ventricular volumes, native T1, T2, and extracellular volume fraction (ECV) were collected. Image analysis was performed using cvi42 version 5.12 (Circle Cardiovascular Imaging Inc., Calgary, AB, Canada). PET images were also reviewed for pathological extra-cardiac FDG uptake, and available clinical and imaging data were assessed for evidence of systemic inflammatory or infiltrative disease, including cardiac sarcoidosis.

### 2.4. Statistical Analysis

Continuous variables are presented as median and interquartile range, and categorical variables as number and percentage. Given the small sample size, PET-positive and PET-negative groups were compared using two-sided Mann–Whitney tests for quantitative PET parameters. Exact permutation *p*-values were calculated to account for the small groups and tied observations. Analyses were exploratory, and unadjusted *p*-values are reported. A two-sided *p*-value < 0.05 was considered statistically significant in this hypothesis-generating analysis. Given the multiple quantitative PET parameters assessed, a Bonferroni-adjusted significance threshold of *p* < 0.0083 (0.05/6) was additionally considered as a conservative sensitivity analysis. The study is reported in accordance with the Strengthening the Reporting of Observational Studies in Epidemiology (STROBE) recommendations. Statistical analyses were performed using R version 4.5.1 (R Foundation for Statistical Computing, Vienna, Austria). ChatGPT-5.5 (OpenAI, San Francisco, CA, USA) was used solely for English-language editing, including grammar, wording, and readability. All suggested changes were reviewed and approved by the authors, who take full responsibility for the final manuscript.

## 3. Results

### 3.1. Study Population

**Figure 1:**
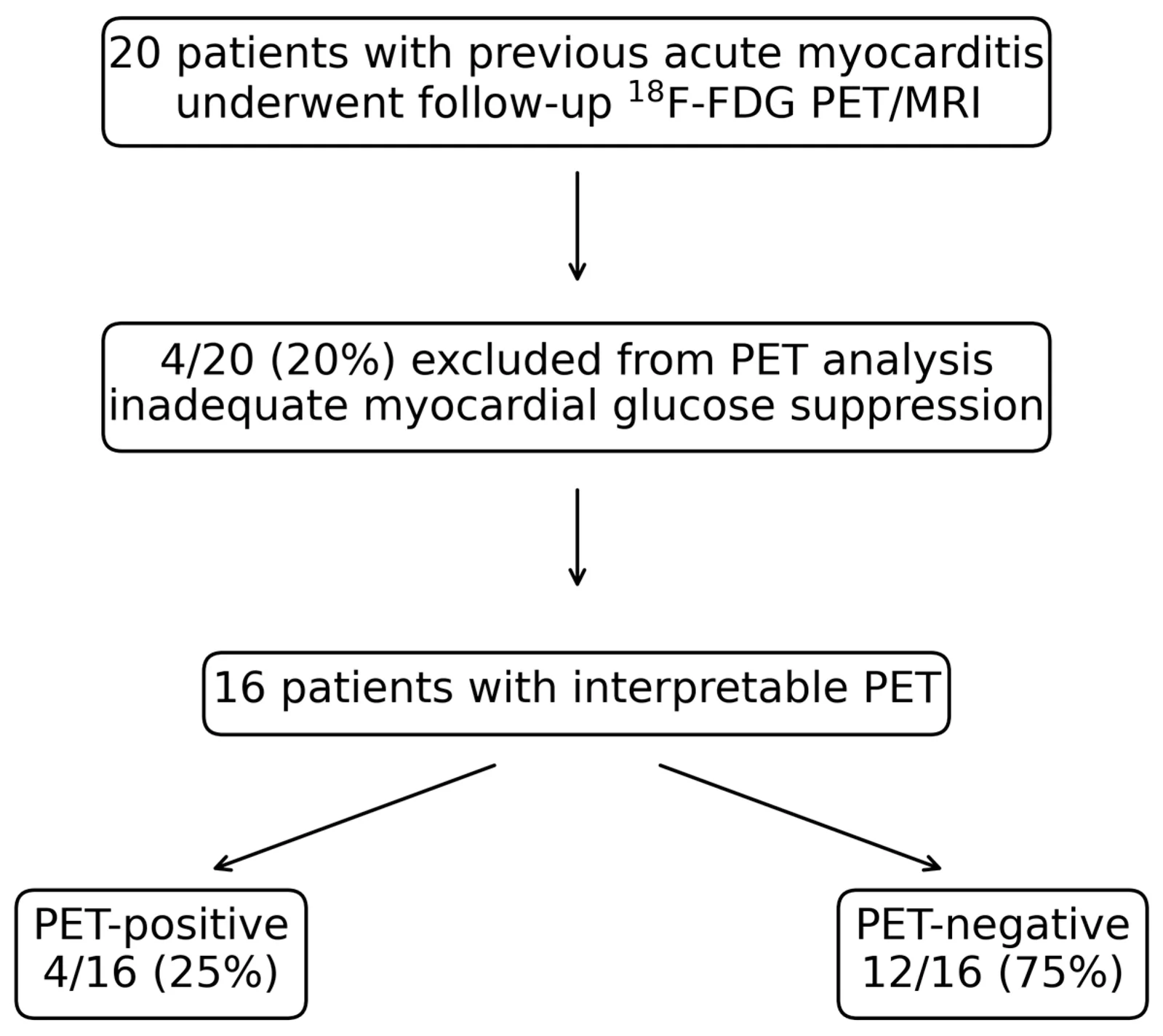
Study flow diagram.

Twenty patients underwent follow-up PET/MRI at a median of 202 days (IQR, 173–221) after the acute episode. Median age was 44 years (IQR, 29.8–56.5), and 40% were women. Four examinations (20%) were non-diagnostic for PET because of diffuse intense myocardial FDG uptake related to inadequate myocardial glucose suppression, leaving 16 evaluable patients (Table 1; Figure 1). Among the four patients with inadequate myocardial glucose suppression, none had diabetes, and median BMI was 24.5 kg/m^2^ (range, 21–28 kg/m^2^).

### 3.2. PET/MRI Findings

**Figure 2:**
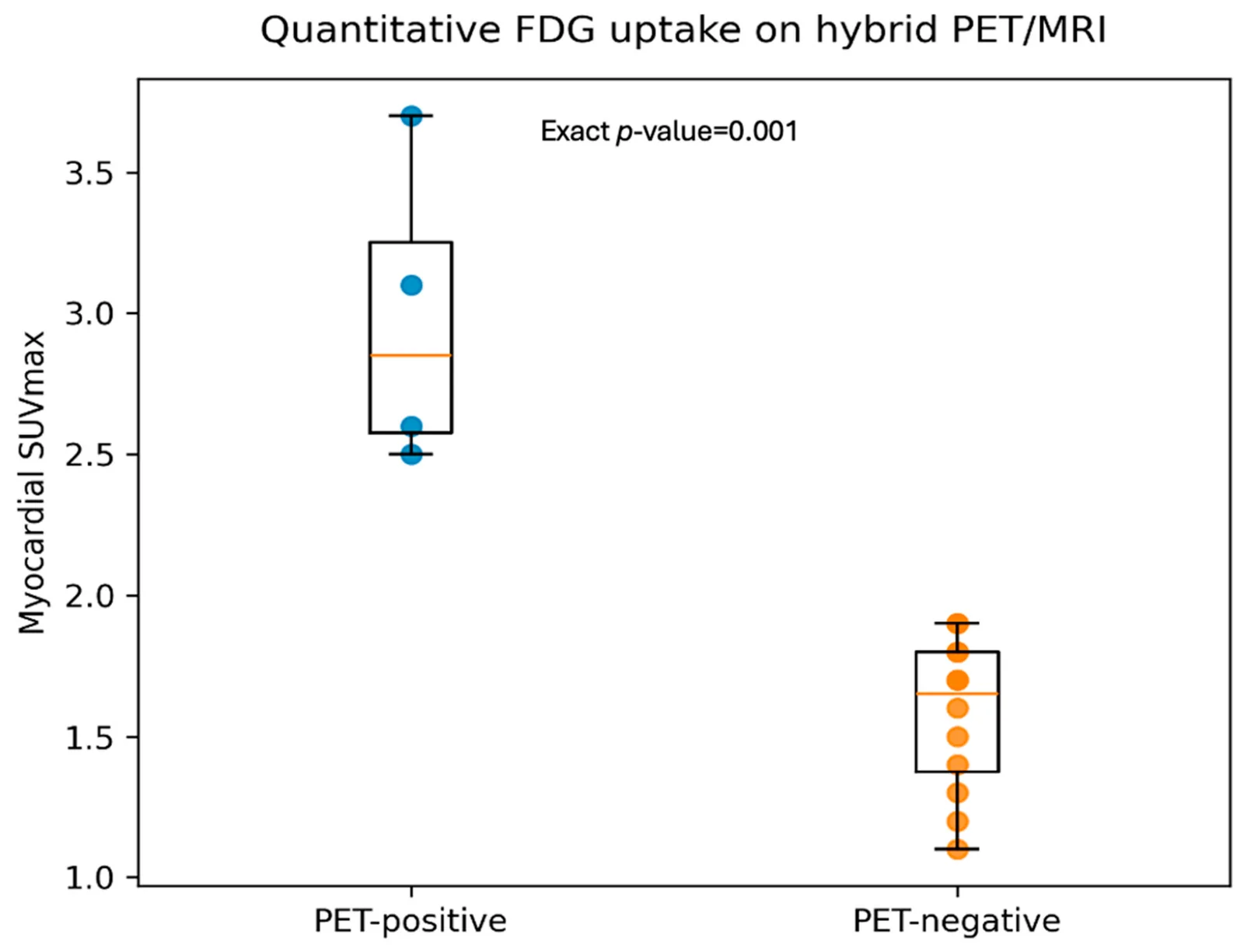
Myocardial SUVmax according to PET status on integrated hybrid PET/MRI. Individual patient values are overlaid on boxplots. PET-positive group, *n* = 4; PET-negative group, *n* = 12.

Four of the 16 evaluable patients (25%) remained PET-positive approximately 6 months after the index episode, with focal myocardial FDG uptake on the integrated PET/MRI examination. The median number of involved segments was three (IQR, 1.75–4). The lateral wall was involved in three patients and the anterior wall in one. Persistent LGE was present in all four PET-positive patients and involved a median of two segments (IQR, 1–3). In all four patients, focal FDG uptake spatially co-localised with areas of persistent LGE. In two patients, the extent of LGE exceeded that of focal FDG uptake. Myocardial SUVmax was higher in PET-positive than PET-negative patients [2.85 (IQR, 2.58–3.25) vs. 1.65 (IQR, 1.38–1.80), exact *p* = 0.001], as was SUVmean [1.65 (IQR, 1.60–1.75) vs. 1.00 (IQR, 0.90–1.10), exact *p* = 0.001] (Table 2; Figure 2). Using a conservative Bonferroni-adjusted threshold for the six quantitative PET parameters (adjusted significance threshold, *p* < 0.0083), the differences in myocardial SUVmax, SUVmean, myocardium-to-blood-pool ratio, and myocardium-to-aorta ratio remained significant.

**Figure 3:**
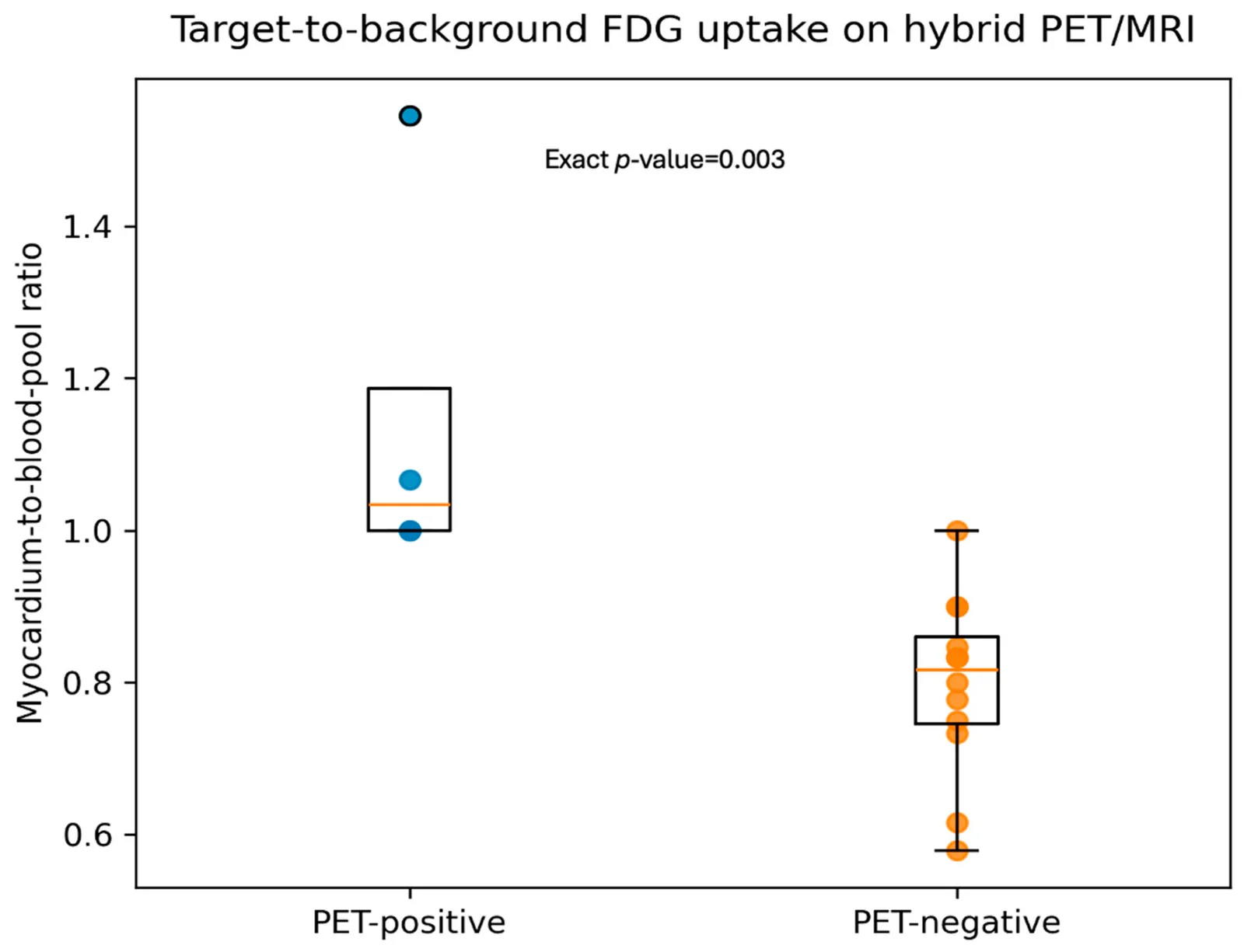
Myocardium-to-blood-pool FDG uptake ratio according to PET status on integrated hybrid PET/MRI. Individual patient values are overlaid on boxplots. PET-positive group, *n* = 4; PET-negative group, *n* = 12.

The myocardium-to-blood-pool ratio was also higher [1.03 (IQR, 1.00–1.19) vs. 0.82 (IQR, 0.75–0.86), exact *p* = 0.003], as was the myocardium-to-aorta ratio in patients with available aortic measurements [1.03 (IQR, 1.00–1.13) vs. 0.78 (IQR, 0.69–0.82), exact *p* = 0.002] (Figure 3). Background blood-pool and aortic activity did not differ significantly between groups. Importantly, none of the four PET-positive patients had myocardial oedema on simultaneously acquired T2 mapping, whereas all four had persistent LGE. T2 mapping encompassed the myocardial regions demonstrating focal FDG uptake in all four PET-positive patients.

**Figure 4:**
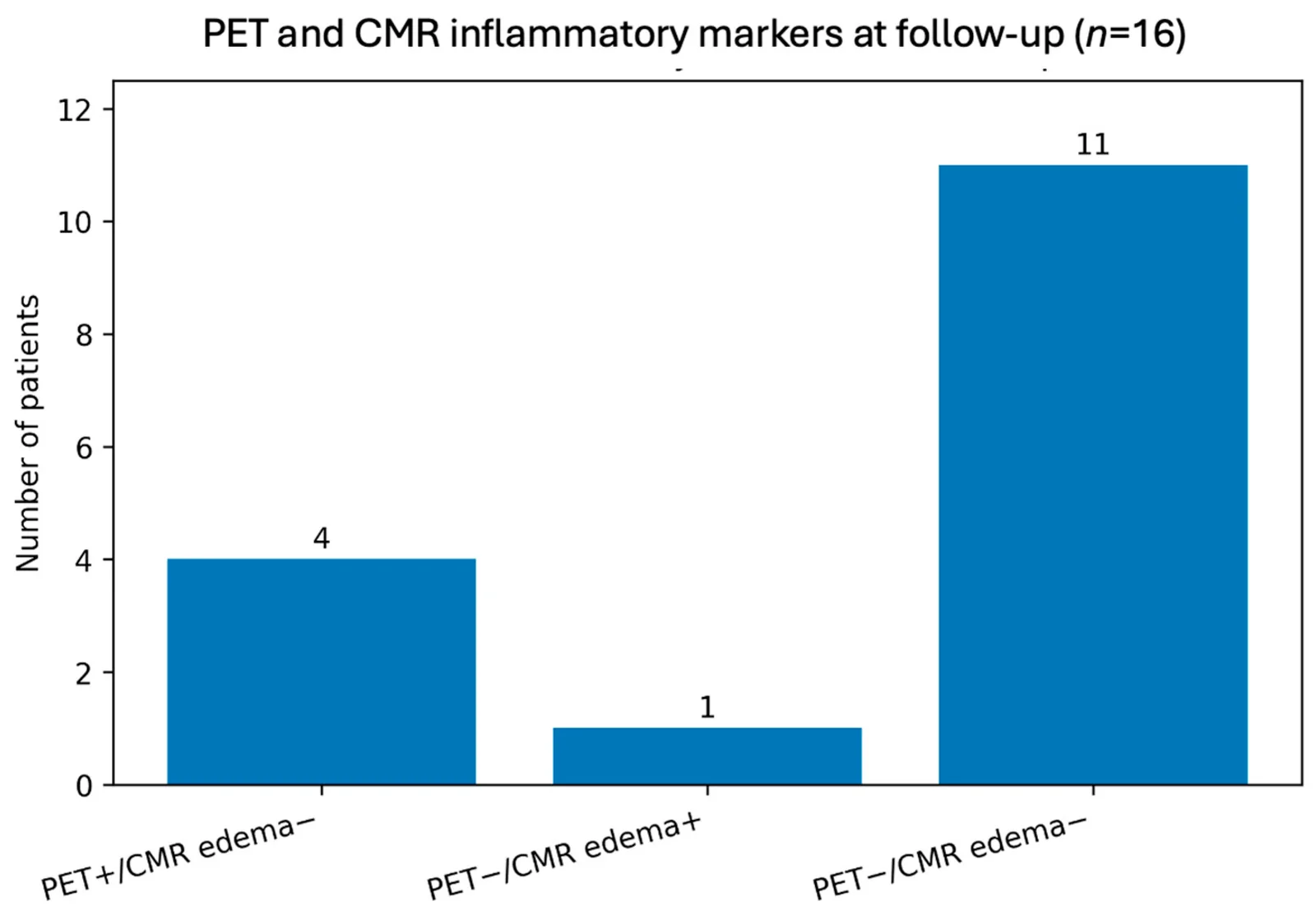
Distribution of PET and CMR inflammatory-marker patterns at follow-up. CMR positivity refers to persistent myocardial oedema on T2 mapping.

Among the 12 PET-negative patients, 11 (92%) had no myocardial oedema on CMR, whereas one patient had persistent oedema involving six segments. Thus, PET and CMR markers of persistent inflammatory activity were discordant in five of 16 evaluable patients (31%) (Figure 4).

**Figure 5:**
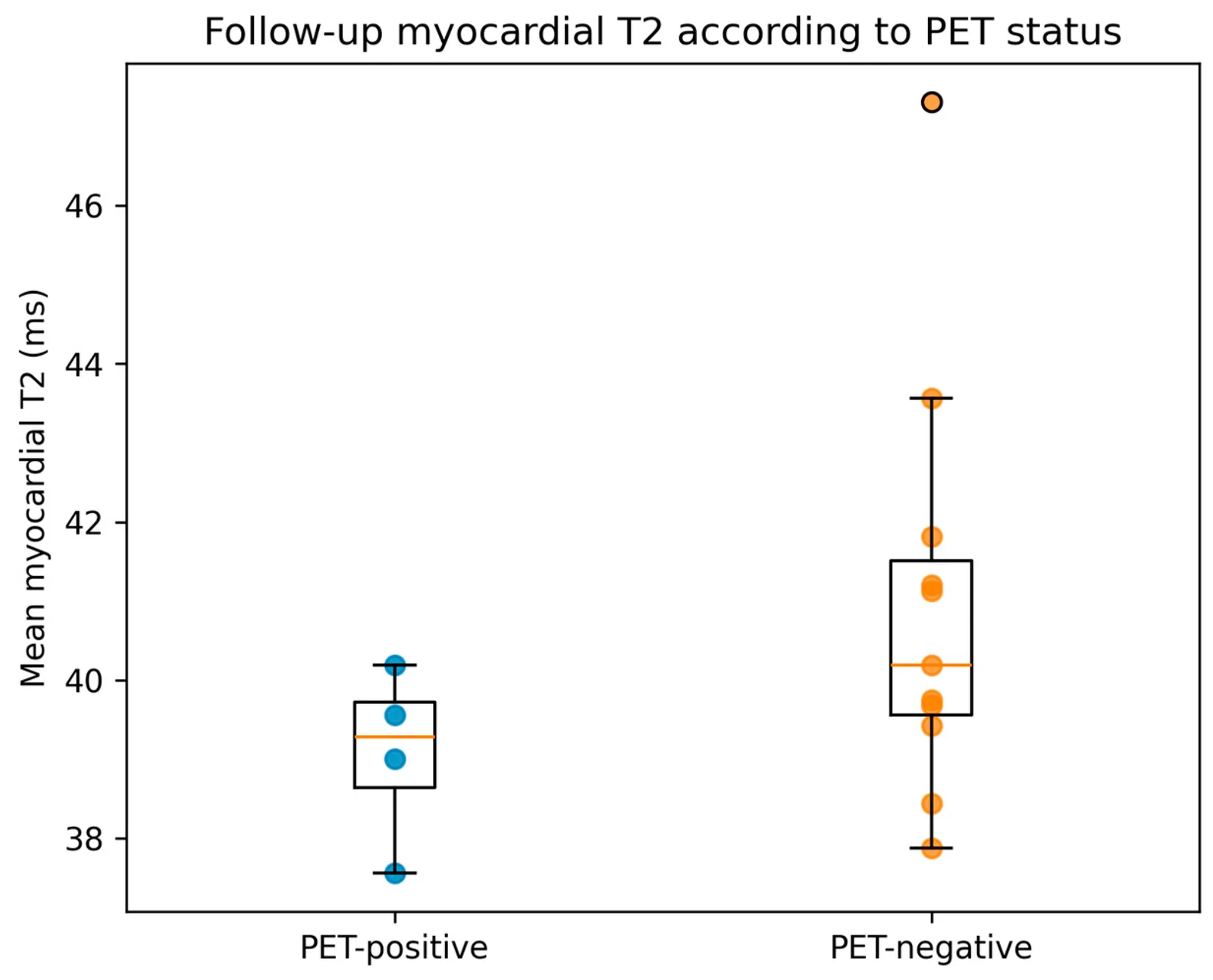
Mean myocardial T2 values according to PET status at follow-up. Individual values are overlaid on boxplots. PET-positive group, *n* = 4; PET-negative group, *n* = 12.

No significant differences in biomarkers, ventricular function, or acute-stage CMR parameters were detected between PET-positive and PET-negative patients; however, these exploratory comparisons were markedly underpowered given the small sample size *(n* = 4 vs. *n* = 12) (Supplementary Table S1). At follow-up, mean myocardial T2 was lower in PET-positive than PET-negative patients [39 (37–40) vs. 41 (39–42) ms, *p* = 0.04], whereas LVEF, ventricular volumes, native T1, and ECV did not differ significantly (Supplementary Table S2; Figure 5). T. Mean myocardial T2 refers to the per-patient average across the analysed myocardial segments, subsequently summarised as the group median (IQR). Both group medians were within the normal range; the absolute difference was small, and the nominal *p*-value of 0.04 should therefore be interpreted cautiously given the multiple exploratory CMR comparisons and the small sample size.

No patient showed imaging or clinical findings supporting an alternative systemic inflammatory or infiltrative diagnosis, including cardiac sarcoidosis.

## 4. Discussion

The principal finding of this pilot hybrid-imaging study is that four of 16 evaluable patients (25%) remained PET-positive approximately 6 months after acute myocarditis, with quantitatively higher myocardial FDG uptake than PET-negative patients, despite the absence of oedema on simultaneously acquired CMR. The integrated nature of PET/MRI is central to this observation: metabolic activity, T2-based oedema, LGE, and ventricular function were assessed during the same examination, limiting temporal mismatch between modalities. The finding of persistent focal metabolic activity in one-quarter of evaluable patients is noteworthy despite the small sample size and should be considered hypothesis-generating.

CMR is highly effective during the acute phase of myocarditis, particularly when oedema-sensitive imaging and non-ischemic injury criteria are combined [7]. Its interpretation later in the disease course is more complex. Oedema may normalise during recovery, whereas LGE can persist and may predominantly reflect residual injury or fibrosis rather than active inflammation. This creates a potential diagnostic gap when ongoing inflammatory activity is suspected.

^18^F-FDG PET offers a complementary metabolic approach. Nensa et al. reported the feasibility of integrated PET/MRI in acute myocarditis, with substantial agreement between PET and CMR inflammatory findings [10]. More recent literature supports the idea that the two techniques interrogate different components of myocardial inflammation. In suspected immune checkpoint inhibitor myocarditis, Tong et al. found only moderate agreement between CMR and ^18^F-FDG PET and concluded that CMR predominantly informs on oedema and tissue injury, whereas PET provides complementary information on glucose metabolism and inflammatory-cell activity [22]. This conceptual framework is relevant to our cohort, in which four patients showed focal FDG uptake despite normalisation of T2-based oedema markers. Importantly, this discordance should not be interpreted as evidence that FDG PET is more sensitive than CMR for detecting persistent myocardial inflammation. In the absence of contemporaneous biological or histological evidence of active inflammation, no independent reference standard was available. Rather, our findings suggest that FDG uptake and T2-based myocardial oedema may reflect different biological components of the post-acute myocardial response and may not resolve synchronously.

Our observations also complement recent hybrid PET/MRI literature. Fogante et al. highlighted the incremental diagnostic contribution of PET/MRI in inflammatory cardiac disease, particularly when standalone findings were equivocal [21]. In their myopericarditis experience, persistent LGE without oedema was frequently accompanied by absent pathological FDG uptake, supporting a non-active phenotype. Our cohort identifies the converse discordant pattern in a subset of patients: four patients remained focally FDG-avid despite absent T2-defined oedema. Together, these findings emphasise that scar/injury, oedema, and metabolic activity may not evolve synchronously and illustrate why simultaneous hybrid PET/MRI may be particularly informative during the post-acute phase. Conversely, one patient showed persistent T2-defined myocardial oedema involving six segments despite the absence of pathological myocardial FDG uptake. This reverse discordant pattern further illustrates that FDG uptake and CMR oedema should not be considered interchangeable markers. Whether this finding reflects differences in the temporal evolution of myocardial abnormalities, distinct underlying biological processes, or limitations in the sensitivity of either imaging marker cannot be determined from the present study.

Persistent metabolic abnormalities beyond the acute phase have also been observed in longitudinal PET/MRI studies. Marschner et al. reported residual evidence of myocardial inflammation on FDG PET/MRI at 2-month follow-up in 2 of 17 participants who had acute myocarditis at baseline [23]. Although that population and clinical context differed from ours, these data support the biological plausibility that metabolic inflammatory activity may persist after clinical recovery and after conventional CMR abnormalities have improved.

Patient preparation is another important limitation of cardiac FDG imaging. Four of 20 examinations (20%) were non-diagnostic because of inadequate myocardial suppression. This rate emphasises the importance of rigorous metabolic preparation. Contemporary PET/MRI protocols may use more prolonged carbohydrate restriction; for example, Fogante et al. used a 72 h carbohydrate-free, high-fat diet before imaging [21]. Differences in preparation strategies should be considered when comparing studies and when designing future prospective protocols.

Despite these limitations, simultaneous PET/MRI provides an attractive framework for studying post-myocarditis biology because ventricular function, myocardial tissue characteristics, scar, and glucose metabolism can be assessed in the same examination. Recent systematic and practical-review literature also emphasises the potential value of hybrid imaging in clinically equivocal inflammatory cardiac disease [20,21]. Future prospective studies should incorporate standardised metabolic preparation, quantitative PET metrics such as SUVmax and target-to-background ratios, comprehensive CMR mapping coverage, contemporaneous biomarkers of myocardial injury and systemic inflammation, including inflammatory cytokine profiling, and clinical endpoints to determine whether persistent FDG uptake reflects ongoing myocardial inflammation and predicts symptoms, arrhythmias, ventricular dysfunction, recurrent myocarditis, or adverse remodelling.

From a clinical perspective, the observation that four patients remained metabolically positive despite absent CMR oedema raises the possibility that hybrid PET/MRI could identify a post-acute phenotype not captured by oedema-sensitive CMR alone. At present, these findings should not alter routine surveillance because the prognostic meaning of persistent focal FDG uptake is unknown. However, simultaneous PET/MRI may be particularly informative in selected patients with persistent symptoms, recurrent biomarker elevation, arrhythmias, suspected immune-mediated myocarditis, or otherwise equivocal follow-up CMR. Prospective studies linking this hybrid-imaging phenotype to clinical outcomes are needed before treatment or surveillance strategies can be based on PET positivity.

### Limitations

This study has several limitations. It was retrospective, single-centre, and included only 20 patients, with 16 interpretable PET examinations and four persistently PET-positive cases. Histological confirmation was not systematically available, so focal FDG uptake cannot be equated with histologically proven active myocarditis. Accordingly, persistent focal FDG uptake should be interpreted as an imaging phenotype rather than definitive evidence of histologically active post-myocarditis inflammation. In addition, systematic contemporaneous measurements of myocardial injury and inflammatory biomarkers at the time of follow-up PET/MRI, including high-sensitivity troponin, C-reactive protein, NT-proBNP, and circulating inflammatory cytokines, were not available. Consequently, the biological significance of persistent focal FDG uptake and its relationship with ongoing myocardial inflammation cannot be definitively established. Four PET examinations were non-diagnostic because of inadequate myocardial glucose suppression. Thus, focal FDG positivity was observed in 4/16 (25%) evaluable examinations, corresponding to 4/20 (20%) of the overall cohort. Exclusion of non-diagnostic examinations may have introduced selection bias and highlights the practical limitations of myocardial FDG suppression in this setting. Image interpretation was jointly performed by an experienced cardiovascular radiologist and a nuclear physician, but no independent second reading or formal inter-observer reproducibility analysis was available. Quantitative PET comparisons are exploratory because of the small sample size and multiple parameters assessed. In addition, absolute myocardial SUV measurements may be influenced by spatial resolution and partial-volume effects, particularly in relatively thin myocardial walls, as well as by MR-based attenuation correction. Finally, comprehensive longitudinal clinical follow-up is not currently available for the four persistently PET-positive patients. Consequently, we cannot determine whether this hybrid PET/MRI phenotype is associated with subsequent symptoms, arrhythmias, recurrent myocarditis and ventricular dysfunction.

## 5. Conclusions

Integrated ^18^F-FDG PET/MRI demonstrated persistent focal metabolic abnormalities in four of 16 evaluable patients (25%) approximately 6 months after acute myocarditis. These patients had higher quantitative myocardial FDG uptake despite absent oedema on simultaneously acquired CMR, while LGE persisted in all four. Although the sample is small and the clinical significance of this phenotype cannot currently be established, the simultaneous hybrid assessment highlights a potentially important dissociation between myocardial metabolism, oedema, and residual injury. Prospective hybrid PET/MRI studies incorporating standardised metabolic preparation, quantitative PET metrics, comprehensive CMR mapping, contemporaneous biological characterisation, clinical outcomes, and rhythm follow-up are warranted.

## Figures and Tables

**Table 1:** Main study characteristics and follow-up findings.

Variable	Value
Patients undergoing PET/MRI	20
Follow-up interval	202 days (IQR 173–221)
Non-diagnostic PET due to failed suppression	4/20 (20%)
Evaluable PET examinations	16
Focal PET-positive	4/16 (25%)
PET-positive with CMR oedema	0/4
PET-negative with CMR oedema	1/12 (8%)
Discordant PET/CMR inflammatory markers	5/16 (31%)

**Table 2:** Quantitative PET parameters according to PET status.

Parameter	PET-Positive (*n* = 4)	PET-Negative (*n* = 12)	*p*-Value
Myocardial SUVmax	2.85 (2.58–3.25)	1.65 (1.38–1.80)	0.001
Myocardial SUVmean	1.65 (1.60–1.75)	1.00 (0.90–1.10)	0.001
Blood-pool SUV	1.55 (1.40–1.68)	1.20 (1.08–1.35)	0.166
Myocardium/blood-pool ratio	1.03 (1.00–1.19)	0.82 (0.75–0.86)	0.003
Aortic SUV *	1.55 (1.45–1.68)	1.25 (1.20–1.38)	0.096
Myocardium/aorta ratio *	1.03 (1.00–1.13)	0.78 (0.69–0.82)	0.002

Values are median (IQR). *p*-values are exploratory two-sided exact permutation Mann–Whitney tests; *p*-values are unadjusted. A Bonferroni-adjusted threshold of *p* < 0.0083 was considered as a conservative sensitivity analysis for the six quantitative PET comparisons. * Aortic SUV measurements were available in 15 of the 16 patients with interpretable PET examinations. In one patient, aortic SUV was not assessed because of previous ascending aortic replacement with a supracoronary vascular graft, precluding the use of the ascending aorta as a comparable reference region.

## Data Availability

The data that support the findings of this study are available from the corresponding author upon reasonable request.

## Supplementary Materials

The following supporting information can be downloaded at: https://www.mdpi.com/article/10.3390/diagnostics16182941/s1, Table S1: Baseline characteristics according to follow-up PET status in patients with interpretable PET examinations; Table S2: Follow-up CMR parameters according to PET status in patients with interpretable PET examinations.

## Abbreviations

CMR	Cardiovascular Magnetic Resonance
ECV	Extracellular Volume
FDG	Fluorodeoxyglucose
IQR	Interquartile Range
LGE	Late Gadolinium Enhancement
LV	Left Ventricle/Left Ventricular
LVEF	Left Ventricular Ejection Fraction
MRI	Magnetic Resonance Imaging
PET	Positron Emission Tomography
PET/MRI	Positron Emission Tomography/Magnetic Resonance Imaging
SUV	Standardised Uptake Value
SUVmax	Maximum Standardised Uptake Value
SUVmean	Mean Standardised Uptake Value
T1	Longitudinal Relaxation Time
T2	Transverse Relaxation Time
TBR	Target-to-Background Ratio
