# Supplementary material for: Persistent Focal Myocardial 18F-FDG Uptake Discordant with CMR Oedema on Hybrid PET/MRI Six Months After Acute Myocarditis
Source: Diagnostics (Basel). 2026 Sep 11;16(18):2941. doi: 10.3390/diagnostics16182941 (PMC13605909; doi:10.3390/diagnostics16182941)
Supplement: Supplementary file 1 [file diagnostics-16-02941-s001.zip › diagnostics-4525463-supplementary.pdf]

Supplementary Table S1. Baseline characteristics according to follow-up PET status in patients with interpretable PET examinations.

Baseline characteristics	PET-positive (*n*=4)	PET-negative (*n*=12)	*p*-value
**Acute-stage CMR parameters**			
LVEF, %	62 (52–69)	56 (31–64)	0.30
LVEDV index, mL/m^2	68 (49–81)	71 (57–92)	0.50
LVESV index, mL/m^2	36 (20–43)	37 (23–44)	0.50
Native myocardial T1, ms	1063 (1050–1075)	1160 (1079–1208)	0.19
Myocardial T2, ms	51 (50–52)	52 (49–58)	0.70
ECV, %	28 (27–29)	31 (27–39)	0.40
**Biomarkers**			
hs-Troponine, ng/L	554 (205–1010)	621 (120–1150)	0.41
CRP, mg/L	65 (15–84)	47(8–75)	0.11
NT-pro BNP pg/mL	311 (153–622)	376 (132–710)	0.64

Data are presented as median (interquartile range) or n (%), as appropriate. Comparisons were performed between the 4 PET-positive and 12 PET-negative patients with interpretable follow-up PET examinations. Given the small sample size, these comparisons should be considered exploratory.

Supplementary Table S2. Follow-up CMR parameters according to PET status in patients with interpretable PET examinations.

CMR parameters	PET-positive (*n* = 4)	PET-negative (*n* = 12)	*p*-value
LVEF, %	59 (41–72)	60 (41–72)	0.76
LVEDV index, mL/m^2	62 (46–69)	70 (63–89)	0.13
LVESV index, mL/m^2	24 (19–27)	27 (20–59)	0.25
Native myocardial T1, ms	1210 (1193–1245)	1241 (1228–1287)	0.07
Mean myocardial T2, ms	39 (37–40)	41 (39–42)	0.04
Mean ECV, %	26 (22–27)	28 (26–30)	0.07

Data are presented as median (interquartile range). Comparisons were performed between patients with interpretable follow-up PET examinations. CMR, cardiovascular magnetic resonance; ECV, extracellular volume fraction; LVEDV, left ventricular end-diastolic volume; LVEF, left ventricular ejection fraction; LVESV, left ventricular end-systolic volume; PET, positron emission tomography.
